# Vaccination against *Streptococcus pneumoniae* Using Truncated Derivatives of Polyhistidine Triad Protein D

**DOI:** 10.1371/journal.pone.0078916

**Published:** 2013-10-31

**Authors:** Charles D. Plumptre, Abiodun D. Ogunniyi, James C. Paton

**Affiliations:** Research Centre for Infectious Diseases, School of Molecular and Biomedical Science, University of Adelaide, Adelaide, South Australia, Australia; Instituto Butantan, Brazil

## Abstract

Polyhistidine triad protein D (PhtD) has been described as a promising vaccine candidate for use against *Streptococcus pneumoniae*, but there has been a lack of examination of its structure and of which region(s) of the protein are targeted by protective immune responses. In this study, we purified recombinant truncated derivatives of PhtD and examined their secondary structural composition, as well as their capacity to bind antibodies from polyclonal murine serum generated against the full length protein. This allowed the identification of a particularly immunogenic fragment of PhtD, which was also purified and characterised. The truncated derivatives were tested as vaccine antigens in mouse models of pneumococcal sepsis and colonisation, using alum and *E. coli* heat labile toxin B subunit respectively as adjuvants. These experiments revealed that whilst the immunogenic region identified may be a promising candidate to protect against sepsis, the full length PhtD was ineffective at conferring significant protective immunity. These results are significant for the potential for PhtD to be used in novel vaccines, which are currently being tested in clinical trials.

## Introduction

*Streptococcus pneumoniae* is a human pathogen able to cause a wide spectrum of diseases, such as pneumonia, sepsis, meningitis and otitis media, and these result in significant global morbidity and mortality [1]. The most recently licensed vaccines against pneumococcal disease are made up of a protein-capsular polysaccharide conjugates. However, these have serious limitations in terms of cost, serotype coverage and increases in the incidence of disease caused by non-vaccine serotypes post introduction [2]–[4].

As an alternative strategy, efforts have been made to identify and characterise pneumococcal protein antigens that can elicit protective immunity and are conserved amongst serotypes. To date, a large number of candidates have been found using a variety of strategies [5]–[9]. One such family of proteins are the polyhistidine triad (Pht) family [10], which has four members (PhtA, PhtB, PhtD and PhtE) in *S. pneumoniae*. These surface-exposed antigens are characterised by the presence of five to six histidine triad (HxxHxH) motifs and are highly conserved amongst pneumococcal strains [11], [12]. All four members have been shown to elicit protective immunity in mouse models of pneumococcal disease against a number of pneumococcal strains ([11], [13]–[16]; summarized in [10]), although one study reported PhtB and PhtE to be less promising candidates in terms of protection elicited against sepsis compared to pneumolysin toxoid PdB, PspA and PspC, where alum was used as the adjuvant [9]. Immunisation with PhtD with AS02 adjuvant leading to protective immunity in rhesus macaques has also been demonstrated [17], and passive transfer of anti-PhtD antibodies purified from naturally exposed humans was shown to protect mice against lethal challenge [16]. More recently, successful phase I clinical trials using PhtD and alum in vaccine formulations have been reported [18], [19]. The Pht proteins are therefore considered highly promising vaccine candidates.

Attention has focussed specifically on PhtD, due to it showing greater conservation amongst strains tested [12] and superior protection elicited in a nasopharyngeal colonisation model with *E. coli* labile toxin as the adjuvant [16] as compared to the other Pht proteins. At 110 kDa, PhtD is a relatively large surface protein, and is thought to be anchored to the surface via attachment to the cell wall [20]. Indeed, we have recently shown that a three amino acid region near the N-terminus of PhtD (Q27-H28-R29) is essential for surface attachment [21]. It therefore seems highly likely that certain regions of PhtD, and indeed the other Pht proteins, will show a varying degree of exposure on the bacterial surface due to the orientation and topology of the protein and the presence of the capsule layer hindering access to deeper regions. Flow cytometry experiments have indicated that regions near the C-termini of the Pht proteins are more exposed than regions near their N-termini [11], [14]. Additionally, immunisation with a fusion protein consisting of the C-termini of PhtB and PhtE with Quil A adjuvant was found to elicit an improved antibody response compared to when the individual truncated protein components were used to immunise mice [14]. The fusion protein was protective in a lethal pneumonia model and passive transfer of antibodies raised against it were protective in a sepsis model [14]. However, these analyses were far from comprehensive and were not performed for PhtD, the most promising vaccine candidate of the family.

In order to increase understanding of the topology of PhtD as well as the contribution to vaccine efficacy of different regions the protein, a series of truncated derivatives of PhtD were constructed and examined.

## Materials and Methods

### Ethics statement

Five to six week old female CD1 mice were obtained from the Laboratory Animal Services breeding facility at the University of Adelaide. Ethics approval for all experiments was granted by the Animal Ethics Committee of the University of Adelaide (project number S-2013-053); experiments were conducted in compliance with the Australian Code of Practice for the Care and Use of Animals for Scientific Purposes (7^th^ edition, 2004) and the South Australian Animal Welfare Act 1985.

### Cloning, expression and purification of truncated derivatives

Primers with *Hind*III and *Bam*HI restriction sites incorporated were designed to amplify truncated derivatives of *phtD*, which were ligated into the pQE-30, -31 and -32 vectors [Qiagen, VIC, Australia] using T4 DNA ligase according to the manufacturer's instructions [New England Biolabs, Mass, USA]. The resultant His_6_-tagged constructs were used to transform *E. coli* BL21 (DE3) *lpxM*− [22]. Expression and purification via metal affinity chromatography were performed as described previously [8].

### Generation of polyclonal antisera

Purified protein antigens were mixed with Imject® Alum [Thermo Scientific, VIC, Australia] and PBS for 30 min at room temperature (RT). Three mice were immunised intraperitoneally on day 0, 14 and 28. Each dose contained 10 µg of protein and 100 µg alum. Mice were euthanised on day 35 and blood was collected from the posterior vena cava. The blood was allowed to coagulate at RT for 30 min before collection of serum by centrifugation. Equal volumes of serum from each of the three mice were pooled.

### ELISA

Proteins were diluted to 5 µg/ml in TSA buffer (132 mM NaCl, 25 mM Tris-HCl, 0.05% w/v NaN_3_, pH 7.5) and allowed to adsorb onto the wells of Nunc MaxiSorp 96 well trays [Thermo Fisher Scientific] overnight at 4°C. Blocking was performed using 1% w/v bovine serum albumin (BSA) dissolved in PBS for 2 h at 37°C. Serial dilutions of pooled mouse serum were subsequently allowed to bind overnight at 4°C, followed by rabbit anti-mouse alkaline phosphatase conjugated secondary antibody [Bio-Rad, NSW, Australia] diluted 1∶3000 v/v in PBS and incubated for 2 h at RT. Lastly, 4-nitrophenyl phosphate disodium salt hexahydrate [Sigma, NSW, Australia] was diluted in substrate buffer (10% v/v diethanolamine, 1 M MgCl_2_, 0.02% w/v NaN_3_, pH 9.8) to 500 µg/ml and incubated at RT in the tray until a yellow colour developed, at which point trays were read at 405 nm using a Spectramax M2 microplate reader [Molecular Devices, California, USA]. Trays were washed five times with PBS between each step. Titres (dilution of mouse serum resulting in half-maximal response) were determined using Prism [GraphPad Software, California, USA].

### Circular dichroism spectroscopy

Proteins were diluted to 0.15 mg/ml in 10 mM sodium phosphate buffer at pH 7.4. Absorbance in the ultraviolet spectrum was measured using a J-815 Spectropolarimeter [Jasco Analytical Instruments, Maryland, USA] according to the manufacturer's instructions. Calculations of proportions of secondary structural elements were performed using the DichroWeb online analysis tool (available at http://dichroweb.cryst.bbk.ac.uk/html/home.shtml) using the CDSSTR programme.

### Sepsis model experiments

Mice were immunised as described above for generation of polyclonal antisera. For the D39 (serotype 2; NCTC 7466) experiment, equal quantities of each protein by mass were used in the immunisations; for the P9 (a serotype 6A strain) experiment, equimolar amounts were used. On day 35, approximately 100 µl of blood was collected by submandibular bleeding. This was allowed to coagulate at RT for 30 min before collection of serum by centrifugation. Equal volumes of serum from each mouse were pooled for antibody titre analysis. On day 42, mice were injected intraperitoneally with pneumococci of strain D39 (5×10^4^ CFU per mouse) or P9 (2×10^5^ CFU per mouse) and survival times were recorded.

### Colonisation model experiment

Mice were immunised intranasally with 10 µl containing 10 µg of protein and 0.2 µg *E. coli* heat labile toxin B subunit (gift from Tom Duthy, University of Adelaide) on days 0, 14 and 28. On day 42, mice were anaesthetised by intraperitoneal injection of pentobarbital sodium [Nembutal; Rhone-Merieux, QLD, Australia] at a dose of 66 µg/g body weight, then given 6.5×10^6^ CFU of strain D39 intranasally by pipetting onto the nares. After 96 hours, mice were euthanised by CO_2_ asphyxiation and pneumococci from the nasopharynx were enumerated as described previously [23].

### Flow cytometry

Bacteria were grown in Todd-Hewitt broth supplemented with 1% yeast extract to mod-log phase, then were incubated with murine antisera serum for 1 h at 37°C followed by Alexa Fluor® 488 rabbit anti-mouse IgG (H+L) [Life Technologies] for 30 min at 4°C. Three washes with 1 ml PBS were performed between each step and all antibodies were diluted 1∶100 v/v in PBS. Fluorescence measurements from 10,000 events were collected using a BD FACSCanto flow cytometer [BD Biosciences, NSW, Australia]. Data were analysed using the software package FlowJo [Tree Star, Oregon, USA].

### Statistics

Survival times and CFU counts of mice immunised with pneumococcal antigens were compared to those of the adjuvant-only negative control groups using one-tailed Mann-Whitney tests.

## Results

### Design and purification of truncated derivatives

**Figure 1:**
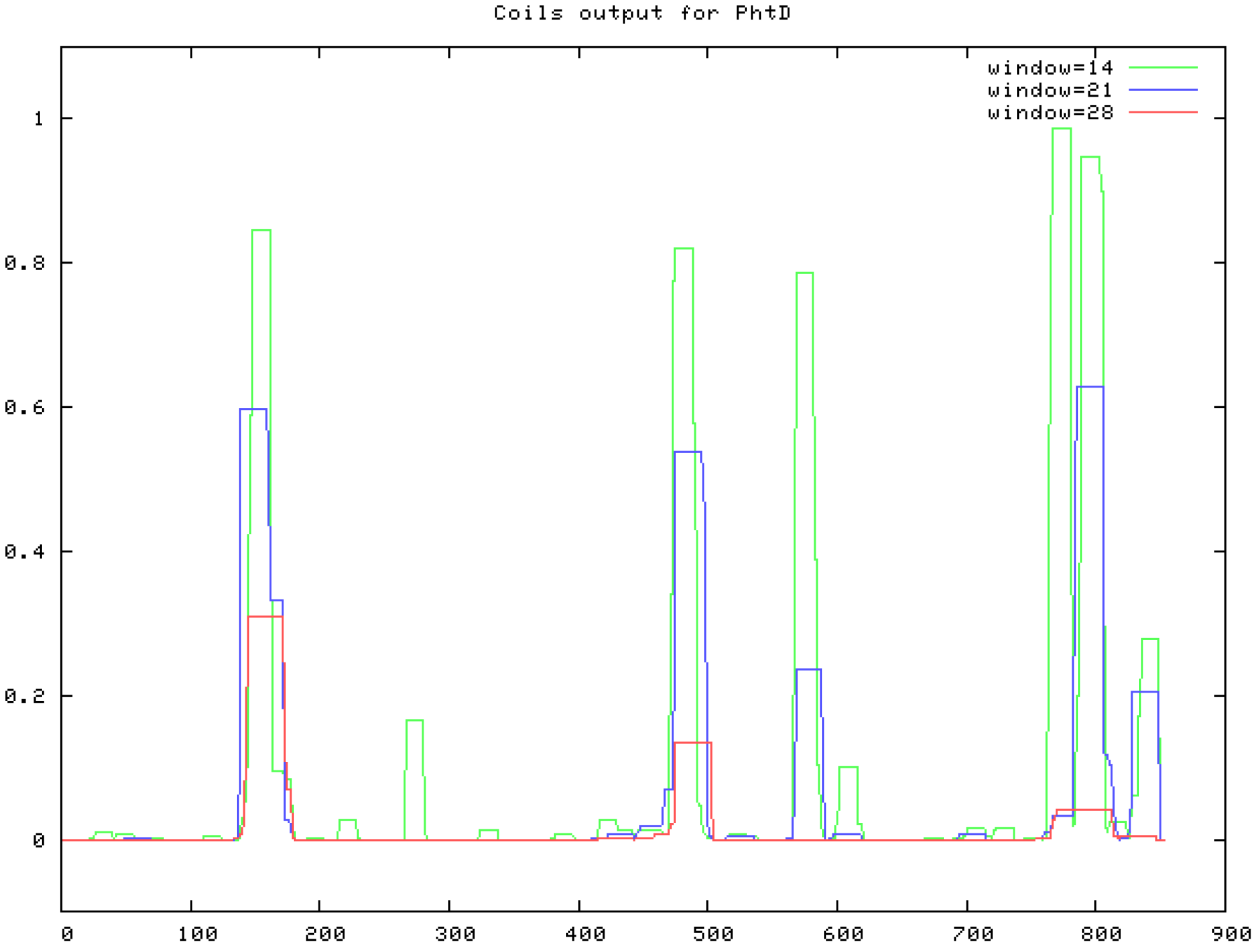
Coils prediction server output for PhtD. The Coils server (http://embnet.vital-it.ch/software/COILS_form.html) was used to compare the amino acid sequence of PhtD to a database of proteins known to form coiled-coils to yield a prediction of which regions (if any) will fold in this conformation. The x-axis represents the position in the protein by amino acid number (starting at the N-terminus) and the y-axis shows how strongly that region is predicted to form a coiled coil. ‘Window’ refers to the width of the amino acid ‘window’ that is scanned at one time.

**Figure 2:**
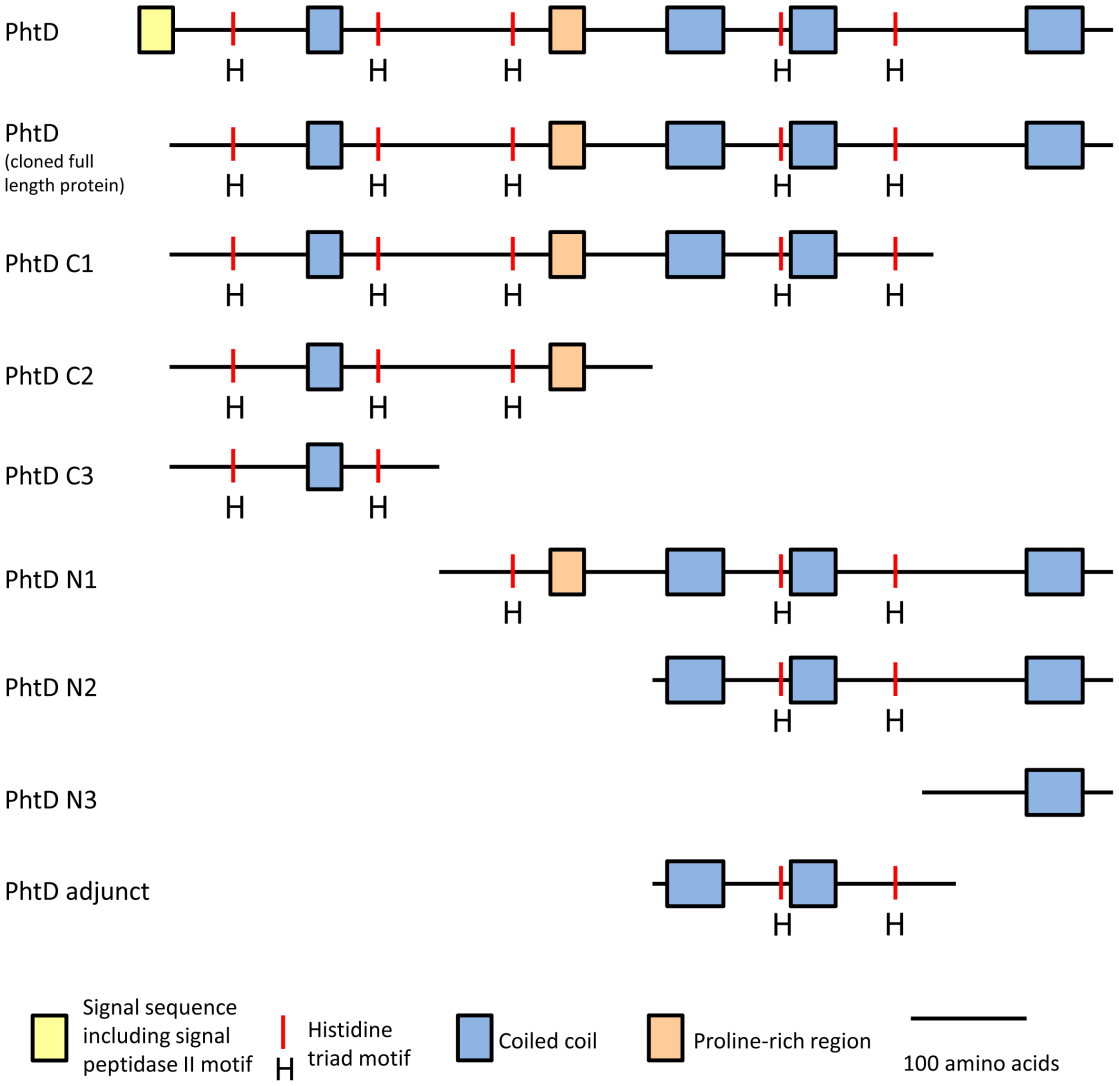
Truncated forms of PhtD used in this study. Proteins are shown with known or predicted domains annotated. The N termini are to the left.

Primers were designed to amplify truncated derivatives of PhtD lacking approximately 200 amino acids and multiples thereof from both the C- and N-terminus of the protein. The first 57 nucleotides, which are predicted to encode the protein's signal peptide, were excluded from all constructs such that this region was not present in the expressed proteins. In order to minimise potential disruption to overall protein structure, the exact positions of the truncations were designed to fall between coiled-coil domains as predicted by the Coils server [24] (see Figure 1). The truncated derivatives that were constructed are represented in Figure 2 and the amino acids included are listed in Table 1.

**Table 1:** List of starting and ending amino acids of PhtD truncated derivatives.

Protein	Starting amino acid	Last amino acid
PhtD C1	Cys20	Phe655
PhtD C2	Cys20	Ser445
PhtD C3	Cys20	Asn247
PhtD N1	Pro248	Gln853
PhtD N2	Asp446	Gln853
PhtD N3	Asp656	Gln853

All purified protein derivatives omit the putative N-terminal signal peptide (amino acids 1 - 19).

**Figure 3:**
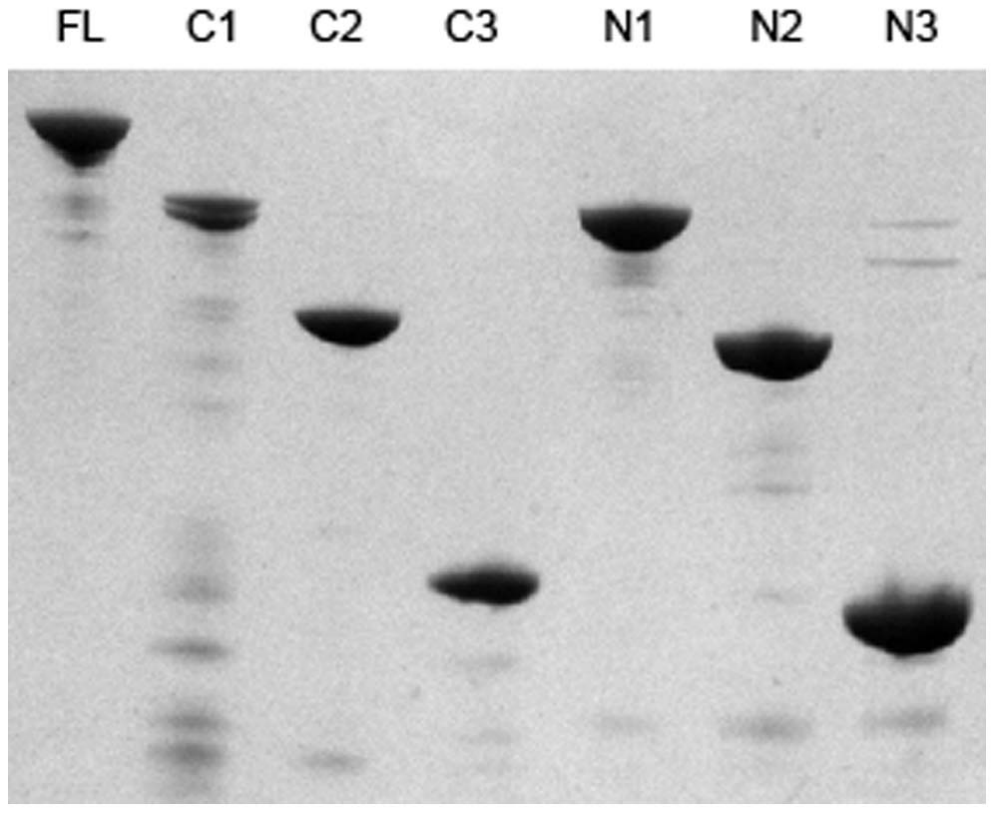
SDS-PAGE analysis of purified truncated derivatives of PhtD. Truncated derivatives of PhtD were analysed by SDS-PAGE using a 12% acrylamide gel and subsequently stained using Coomassie R250. 1 µg of each protein was used. The identity of the protein is indicated above each lane (FL; full length).

The protein products of the cloned genes were expressed in *E. coli* and purified by metal affinity chromatography. All were judged to be >90% pure by Coomassie staining following SDS-PAGE analysis (see Figure 3).

### Antibody binding regions of PhtD

**Figure 4:**
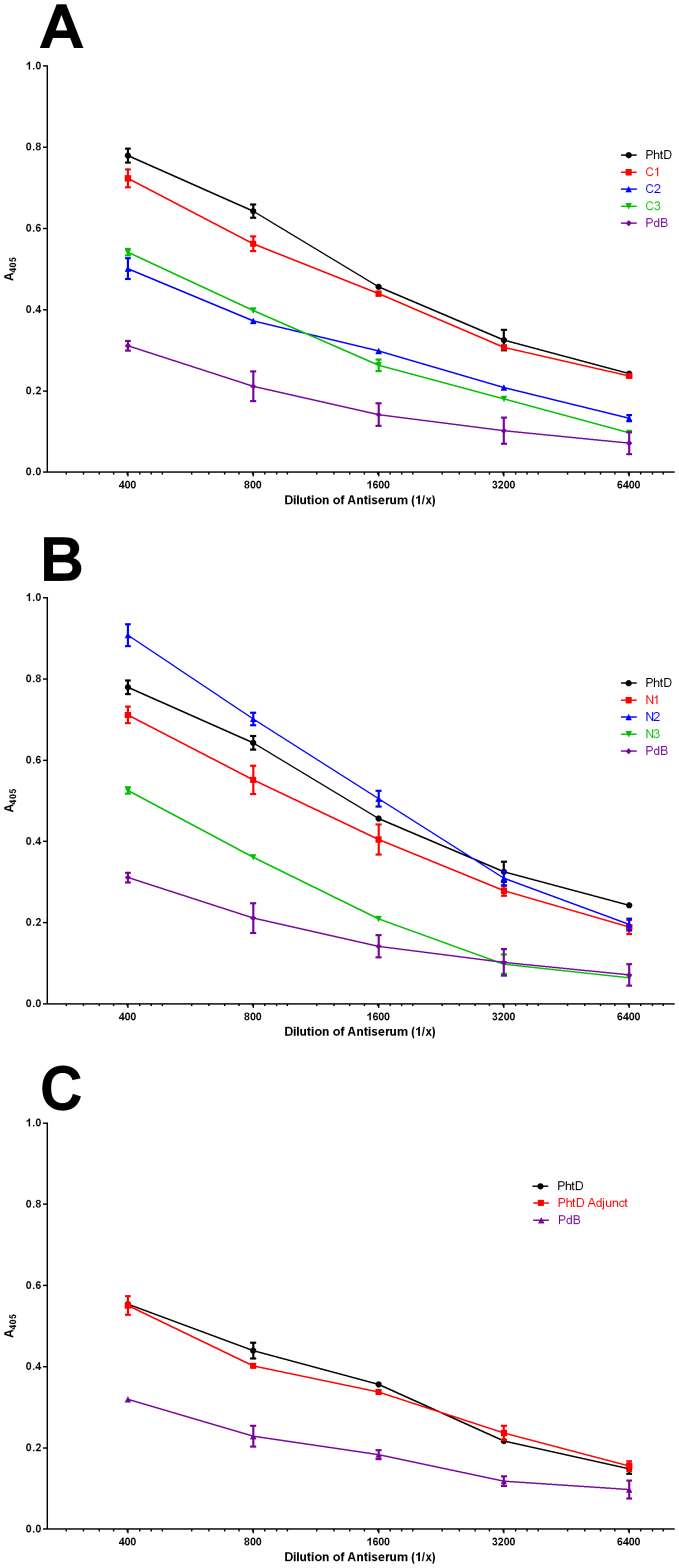
Antibody binding capacity of truncated derivatives of PhtD. ELISA was used to examine binding of anti-PhtD serum to PhtD and truncated derivatives. PdB is an unrelated negative control protein (genetic toxoid derivative of pneumolysin [34]) to which no binding is expected. A: proteins truncated from the C terminus. B: proteins truncated from the N terminus. C: PhtD adjunct protein.

A polyclonal murine antiserum was generated against the full length PhtD protein and ELISAs were performed to examine the extent of binding of the antiserum to the truncated derivative proteins. Results are shown in Figure 4A (truncations from the C terminus) and 4B (truncations from the N terminus). The truncated derivatives can be grouped based on the ability of antibodies in the antisera to bind to them relative to the full length protein: C1, N1 and N2 show high binding whilst C2, C3 and N3 show markedly lower binding. Interestingly, the region of PhtD between Asp446 and Phe655 is common to C1, N1 and N2 (the high antibody binders) but is lacking in C2, C3 and N3 (the low antibody binders), consistent with this region being highly immunogenic.

We therefore cloned, expressed and purified the region consisting of Asp446 to Phe655 (PhtD adjunct) and tested its antibody binding as for the truncated derivatives above (figure 4C). This protein showed a similar level of antibody binding as the full length PhtD, consistent with this region being immunogenic.

### Measurement of protein secondary structural elements by circular dichroism spectroscopy

Circular dichroism (CD) spectroscopy was used to examine the truncated derivatives of PhtD and the full length protein. Data on absorbance in the ultraviolet spectrum were collected and analysed using the CDSSTR algorithm via DichroWeb [25]–[27]. This was performed to provide information on the structure of PhtD, as well as to determine whether the truncated derivatives were disordered in their secondary structural elements, or whether they were folded in a manner representative of the full length protein. Results showing the proportions of each protein predicted to be in alpha-helical, beta-strand, beta-turn or unordered conformations are shown in Table 2. All of the truncated derivatives were found to contain similar proportions in an unordered conformation as the full length protein, consistent with the protein fragments assuming a correctly folded structure. In addition, the results reveal that PhtD has a high proportion of α-helix in the C-terminal half of the protein, since PhtD N1, N2 and the PhtD adjunct contained high proportions of this secondary structural element.

**Table 2:** Circular dichroism spectroscopy analysis of PhtD and truncated derivatives.

Protein	α-helix	β-strand	β-turn	Unordered
PhtD	0.36	0.18	0.18	0.28
PhtD C1	0.25	0.26	0.20	0.30
PhtD C2	0.20	0.26	0.21	0.33
PhtD C3	0.16	0.29	0.21	0.34
PhtD N1	0.49	0.09	0.17	0.25
PhtD N2	0.56	0.07	0.14	0.23
PhtD N3	0.44	0.11	0.20	0.26
PhtD Adjunct	0.51	0.18	0.13	0.18

Results are shown as proportions of each protein predicted to be folded in each of the indicated secondary structural conformations, or predicted to be unordered.

### The PhtD adjunct protein showed statistically significant protection against sepsis caused by strain D39

**Figure 5:**
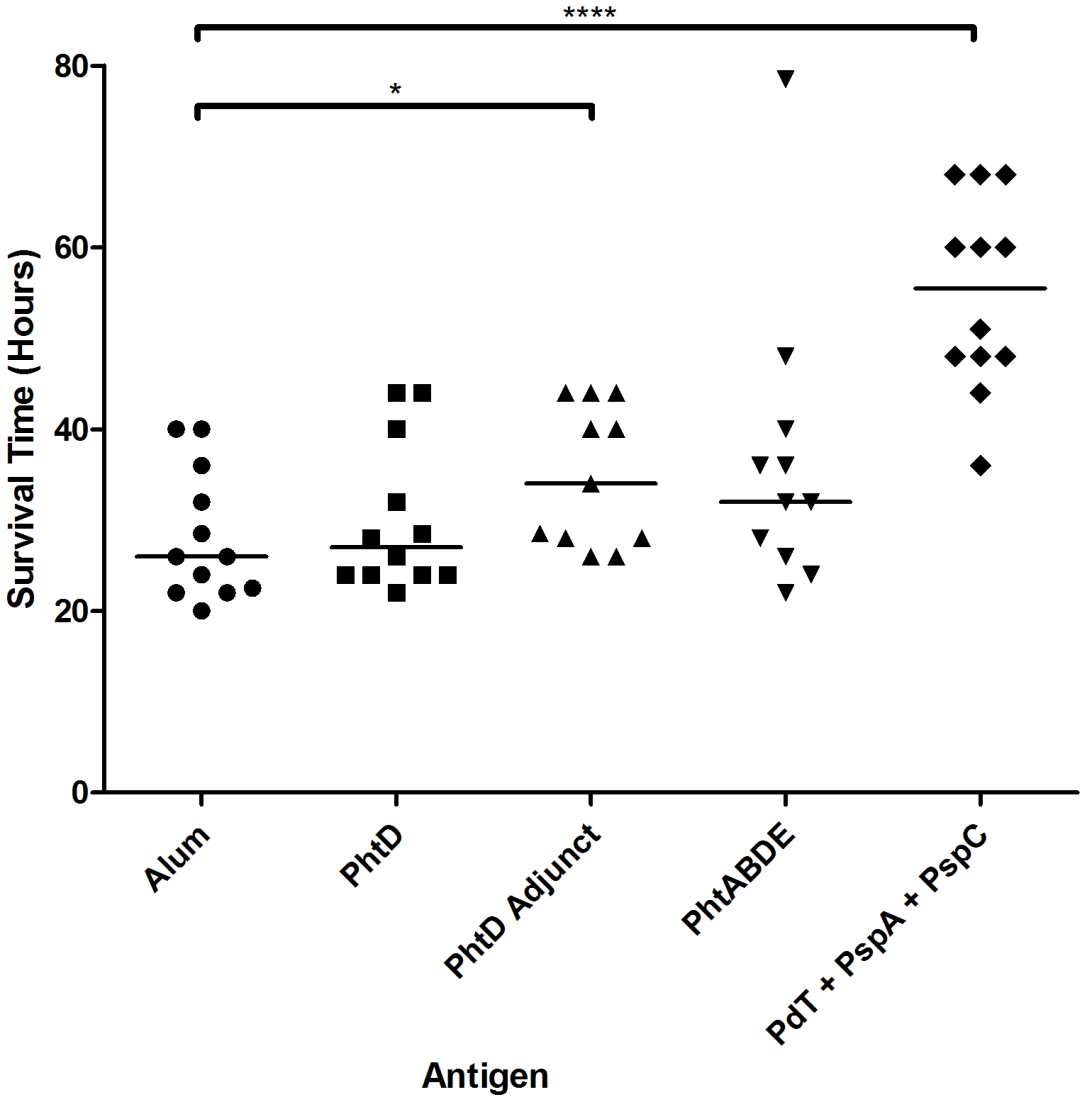
Survival times of mice challenged with D39 after immunisation with the indicated antigens. Median survival times are indicated by horizontal lines. Significant differences from the alum only control group are indicated where present (*, *P*<0.05; ****, *P*<0.0001).

Given that the PhtD adjunct protein bound a high amount of antibody from the polyclonal antiserum, it was of interest to determine whether it could induce protective immunity against systemic pneumococcal disease. Groups of 12 mice were immunised with the indicated proteins and challenged via intraperitoneal injection with 5×10^4^ wild-type pneumococci of strain D39, and survival times were measured (see Figure 5). Only the groups given the PhtD adjunct protein, or the combination of PdT (a genetic toxoid derivative of pneumolysin), PspA and PspC, showed significant increases in median survival time relative to that of the alum control group (*P*<0.05 for the PhtD adjunct and *P*<0.0001 for the combination of PdT, PspA and PspC). Neither the full length PhtD protein nor a combination of PhtA, PhtB, PhtD and PhtD conferred significant protection compared to wild-type. Titres of antibodies specific for each antigen in pooled sera from each group of mice were measured by ELISA and the results are shown in Table 3.

**Table 3:** Antibody titres of pooled sera from immunised mice before challenge with D39.

Mouse Group	Antigen	Titre
PhtD	PhtD	10,000
PhtD Adjunct	PhtD Adjunct	54,000
PhtABDE	PhtA	16,000
PhtABDE	PhtB	17,000
PhtABDE	PhtD	12,000
PhtABDE	PhtE	66,000
PdT + PspA + PspC	PdT	8,800
PdT + PspA + PspC	PspA	3,800
PdT + PspA + PspC	PspC	5,900

Antibody titres were measured via ELISA. Titre was defined as the dilution of mouse serum giving half-maximal absorbance, and was rounded to two significant figures.

### PhtD truncated antigens do not induce protective immunity against colonisation by strain D39

**Figure 6:**
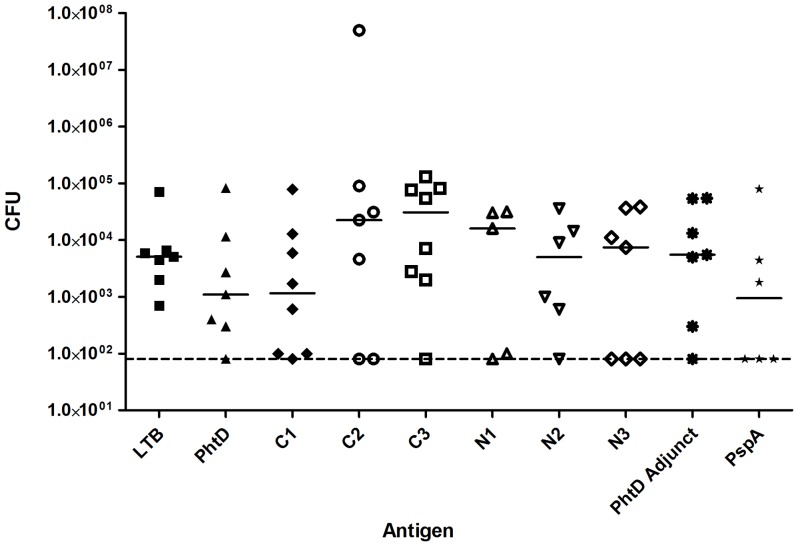
CFU of pneumococci recovered from the nasopharynges of vaccinated mice. After three immunisations with the indicated antigens, mice were challenged intranasally with wild-type D39 pneumococci. After 96 hours, pneumococci from the nasopharynx were enumerated. Median CFU recovered is indicated by the solid lines; the dashed line indicates the limit of detection.

To further investigate the protective roles of different regions of PhtD, protection against colonisation by *S. pneumoniae* was examined by immunising groups of eight mice via the intranasal route with full length PhtD and truncated derivatives using *E. coli* labile toxin B subunit (LTB) as adjuvant. 10 µg of PhtD was given per dose; amounts of truncated derivatives of PhtD were adjusted such that equimolar quantities of these antigens to that of full-length PhtD were given. Control groups were immunised with adjuvant alone (negative control) or with adjuvant and PspA (positive control). Mice were subsequently challenged with strain D39 intranasally under anaesthesia. After 96 hours, mice were euthanized and numbers of pneumococci in the nasopharynx were enumerated. This model was chosen since immunisation with PhtD has previously been shown to significantly reduce pneumococcal colonisation under similar experimental conditions [16]. Results are shown in Figure 6. A small number of mice died from invasive pneumococcal disease before the 96 hours had elapsed; results from these mice were excluded. Whilst modest differences in median CFU recovered can be seen between groups, statistical analysis revealed that none of the groups were significantly different from the adjuvant alone negative control group. This includes the PhtD and PspA positive control groups, which was unexpected since these antigens have previously been shown to confer protective immunity in this model [16].

### PhtD does not confer protective immunity against sepsis caused by strain P9

**Figure 7:**
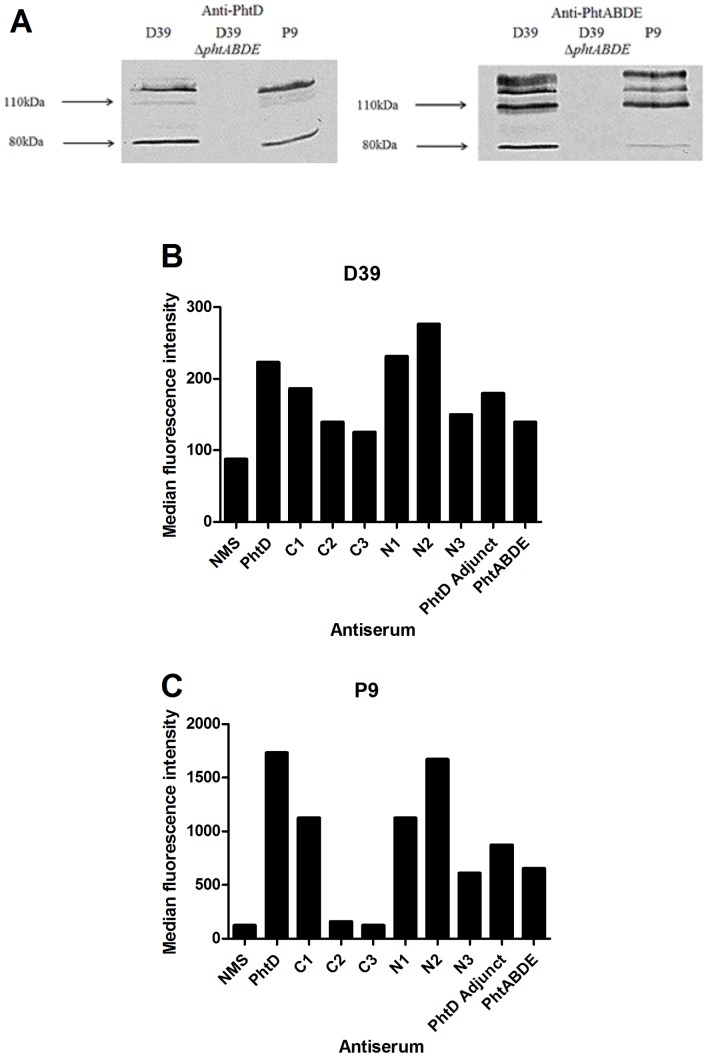
Presence and exposure of Pht proteins in strain P9 compared to D39. A: Western blotting using pneumococcal cell lysates with anti-PhtD (left) or anti-PhtABDE (right) polyclonal sera. Mobilities of molecular size markers are indicated. B and C: detection of PhtD on the surface of D39 (B) and P9 (C) by flow cytometry using antisera generated against PhtD truncated derivatives. NMS, normal mouse serum (negative control). Median fluorescence intensities for 10,000 events are displayed.

To test the effectiveness of immunisation with truncated derivatives of PhtD against disease caused by a different strain from D39, we next examined the clinically relevant serotype 6A strain P9 (recently isolated as part of the Program for Appropriate Technology in Health [PATH] pneumococcal vaccines project). The presence and immunological cross-reactivity of Pht proteins in this strain with Pht proteins of D39 was checked by Western blotting using whole-cell lysates of wild-type P9 and D39, as well as of a D39 Δ*phtABDE* mutant, with anti-PhtD or anti-PhtABDE (from animals immunised with a combination of all four Pht proteins) polyclonal murine sera. As shown in Figure 7A, there were only small differences in the presence or reactivity of bands corresponding to Pht proteins between D39 and P9, implying that murine antibodies generated by immunisation with Pht proteins (which were cloned from D39) were likely to be able to bind P9 in the sepsis model. A band at around 80 kDa was detected in both Western blots; it is not clear whether this represents PhtA (which has a predicted molecular weight of approximately 92 kDa) or a partly degraded form of PhtD.

To examine the surface exposure of Pht proteins, and in particular of the different regions of PhtD, the extent of binding of antisera generated against PhtD or truncated derivatives to wild-type D39 or P9 was measured using flow cytometry (Figure 7B and 7C). This showed that the anti-PhtD and anti-PhtABDE sera bound more readily to P9 than to D39, suggesting that there was greater expression and/or exposure of PhtD on the surface of the former strain, which could translate into more effective PhtD antibody-mediated immunity. Both strains showed similar trends in the relative amounts of binding observed for the antisera generated against the truncated derivatives. The data also indicate that the C terminus of PhtD is more exposed than the N terminus, in line with previous observations, and that the PhtD adjunct region is accessible to exogenous antibody.

**Figure 8:**
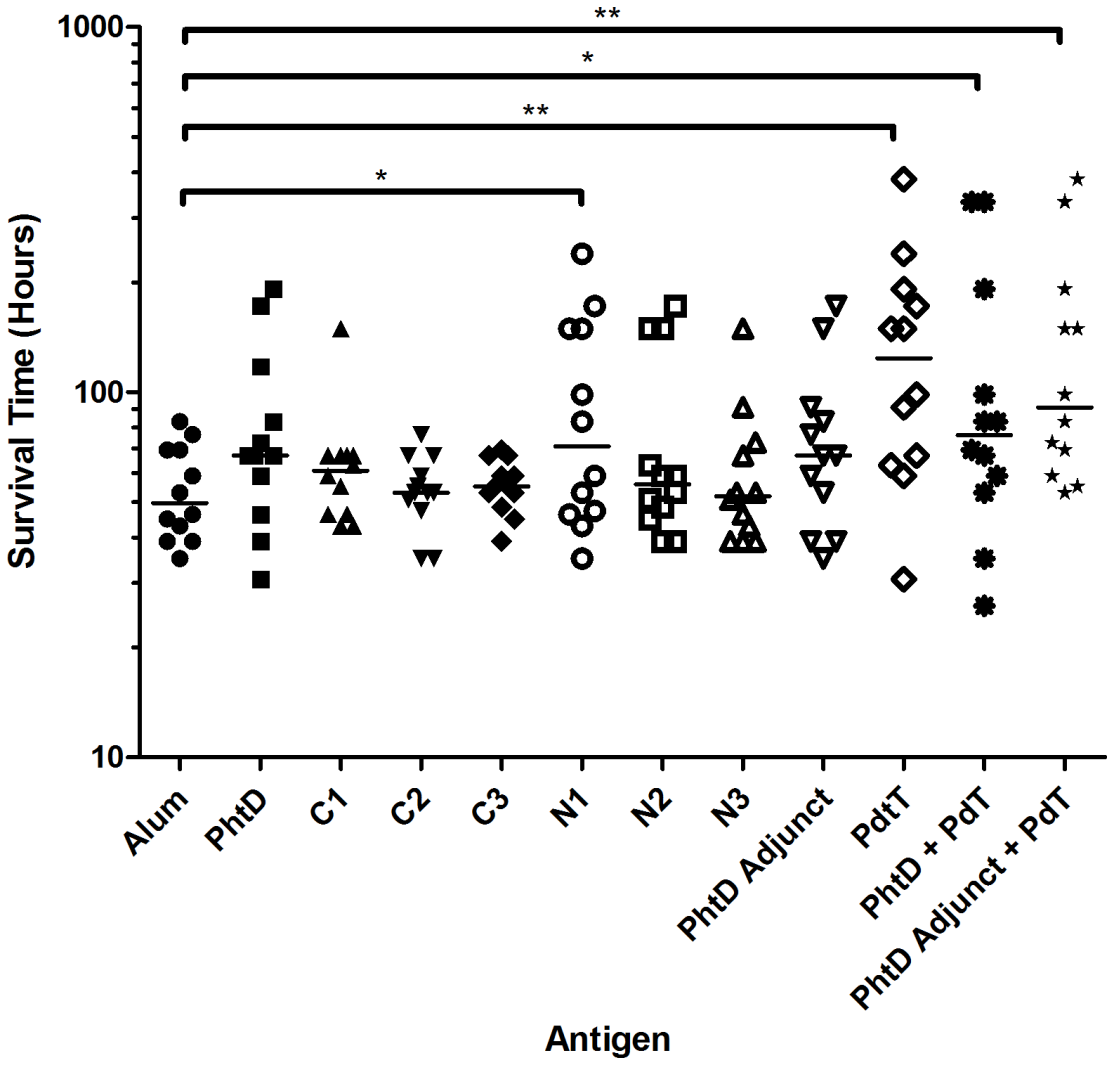
Survival times of mice challenged with P9 after immunisation with the indicated antigens. Median survival times are indicated by horizontal lines. Significant differences from the alum only control group are indicated where present (*, *P*<0.05; **, *P*<0.01).

Lastly, the ability of PhtD and the truncated derivatives to induce protective immunity against sepsis caused by P9 was tested in a mouse model. PdT was used as a positive control immunogen, and combinations of PhtD and PdT or the PhtD adjunct and PdT were also tested to determine whether these antigens could confer additive or synergistic protection. Survival times of the mice were measured and are shown in Figure 8. Whilst marginal increases in median survival time were observed for a number of groups, only groups immunised with PhtD N1, PdT, PhtD + PdT or PhtD adjunct + PdT showed statistically significant differences compared to that of the alum group. Thus, immunisation with full length PhtD did not confer significant protective immunity. Furthermore, PhtD also did not increase the survival time of mice immunised with PhtD + PdT, compared with PdT alone. The same was true of the PhtD adjunct in combination with PdT. Antibody titre measurements (shown in Table 4) from pre-challenge pooled sera demonstrated that, despite the lack of protection, strong IgG responses had been induced, especially for the PhtD adjunct. It was therefore concluded that PhtD is a suboptimal candidate for a protein vaccine component in this model of pneumococcal disease, relative to PdT.

**Table 4:** Antibody titres of pooled sera from immunised mice before challenge with P9.

Mouse Group	Antigen	Titre
PhtD	PhtD	22,000
PhtD + PdT	PhtD	8,400
PhtD C1	PhtD C1	23,000
PhtD C2	PhtD C2	10,000
PhtD C3	PhtD C3	4,700
PhtD N1	PhtD N1	9,700
PhtD N2	PhtD N2	8,600
PhtD N3	PhtD N3	6,400
PhtD Adjunct	PhtD Adjunct	110,000
PhtD Adjunct + PdT	PhtD Adjunct	110,000
PdT	PdT	9,300
PhtD + PdT	PdT	10,000
PhtD Adjunct + PdT	PdT	8,900

Antibody titres were measured via ELISA. Titre was defined as the dilution of mouse serum giving half-maximal absorbance, and was rounded to two significant figures.

## Discussion

PhtD is an important vaccine candidate for use against the pneumococcus and phase II clinical trials are currently underway to assess its potential. In this work, we have explored the structure and immunogenicity of various regions of the protein, as well as its ability to confer protective immunity against both colonisation and invasive disease caused by the pneumococcus.

### Secondary structural elements and immunogenicity of truncated proteins

The circular dichroism spectroscopy data showed that a large proportion of the C-terminal half of PhtD is folded into an alpha helical conformation. This is in concordance with the prediction that coiled-coils, which are composed of alpha helices, are found in this region (Figure 1). The PhtD adjunct protein (Asp446 to Phe655) was found to be highly immunogenic and to contain a large proportion of alpha helix, potentially in a coiled coil arrangement. It is encouraging that such a region should be so immunogenic, since it is also likely that an elongated structure such as a coiled coil would extend out from the pneumococcal surface and would therefore be exposed and available to be bound by antibodies *in vivo*. These two characteristics – immunogenicity and exposure on the surface – are critical for a successful vaccine antigen. It is interesting to note that another leading protein vaccine candidate for the pneumococcus, PspA, also contains coiled-coil motifs that are thought to be immunogenic and exposed [28]. This common structural feature may be necessary to allow these proteins to protrude through the capsule layer and access the extracellular milieu in order to interact with host surfaces and/or carry out their respective functions. However, this could also be an ‘Achilles heel’ that could be targeted by vaccination. There is some apprehension that antibodies against the coiled-coil regions of PspA could be cross-reactive with cardiac myosin, which also forms a coiled-coil structure. However, such concerns can be allayed somewhat by the fact that there are no known associations between pneumococcal infections and autoimmune conditions, even though the majority of adults have significant levels of antibodies to PspA in their serum [29].

The data from the initial ELISAs were largely consistent with the antibody titre data from the D39 and P9 sepsis model experiments (see Tables 3 and 4). These both showed that mice immunised with the PhtD adjunct developed a very high titre of IgG, showing that this region is indeed highly immunogenic.

### Protective immunity elicited by immunisation with truncated proteins in D39 sepsis model

Immunisation with the PhtD adjunct elicited a significant increase in median survival time of mice after challenge with D39, whereas immunisation with the full length protein did not. Since it contains all potential epitopes, it might be expected that the full length protein would either be equal or superior to any truncated forms in terms of vaccine efficacy, but this was not the case. This result may be due to an effect of the dose of protein given. In this experiment, mice were immunised with 10 µg of each protein per dose, which implies that the dose of the adjunct proteins was approximately four-fold greater in molar terms than that of the full length proteins, due to the difference in molecular weight of the full length and adjunct proteins. Consistent with the ELISA data showing that the PhtD adjunct is immunodominant (a high proportion of antibodies in the polyclonal antiserum generated against the full length protein could bind to the PhtD adjunct), the titre of pooled mouse serum from the PhtD adjunct group against the adjunct protein was much greater than that of the PhtD group against the full length protein. This difference in the level of antibody in the mice could account for the increase in survival time, since antibodies against pneumococcal proteins are known to be a major correlate of protective immunity against invasive disease [30], [31].

### Failure to elicit protective immunity in D39 colonisation and P9 sepsis models

Disappointingly, almost no protective effects were seen in either the D39 colonisation or the P9 sepsis models. The result of the former is particularly surprising given that Godfroid *et al*. [16] showed that immunisation with PhtD decreases the bacterial load in the nasopharynx. The two experiments used the same adjuvant, immunisation schedule and challenge strain (D39). There were differences in the challenge dose (7×10^4^ in the published study versus 6.5×10^6^ here) and strain of mice (BALC/c versus CD1) used. BALB/c mice have been shown to be more resistant to pneumococcal infections than several other mouse strains, and this is associated with a more vigorous immune response [32]. However, CD1 mice, which are outbred, were not tested in that study. Recently, another pertinent study has found that significant protection against colonisation is induced by immunisation with either PhtD or PhtD using strain TIGR4 in C57BL/6 mice [33]. The differences in challenge doses and strains of mice and/or pneumococci used in the two previous studies and this report may be responsible for the differences in efficacy of vaccination with PhtD observed.

In the P9 sepsis model, PhtD N1 was the only PhtD derivative that significantly increased survival time compared to the alum control group. This confirms that critical immunogenic epitopes are not located at the N-terminus. However, it cannot be concluded that the PhtD N1 protein was actually more able to induce protective immunity than the full length protein or any truncated derivatives, since there were no statistically significant differences in survival time between the N1 group and any of these other groups. Neither full-length PhtD nor any of the other truncated derivatives conferred significant protective effects compared to the alum control group. This is surprising, given that substantial IgG titres were generated against PhtD and its truncated derivatives, and flow cytometry showed that PhtD on the surface of P9 was highly accessible to these antibodies. It is possible that Pht proteins make a lesser contribution to the pathogenicity of P9 than they do to other strains, which would mean that blockade of their function via antibody binding would have a lesser effect on the course of disease.

When combined with the pneumolysoid PdT, neither full-length PhtD nor the PhtD adjunct increased the median survival time compared to when PdT was given alone. A similar absence of additive protection has previously been reported for combinations of PhtB and PhtE with PspA and another pneumolysin toxoid PdB [9]. Outside of this report, there is a dearth of reports examining immunisation with combinations of Pht and other pneumococcal antigens. In light of the results found here, it will be important to examine this in future studies, since the lack of additive protection could make Pht proteins significantly less attractive as vaccine candidates.

This study has identified a potentially important region in PhtD that is highly immunogenic and surface exposed, but has also cast doubt on the efficacy of PhtD as a vaccine candidate relative to other antigens. If PhtD is to be investigated further as a vaccine candidate, the adjunct region should be considered as a replacement for the full length protein, given its potential to elicit superior antibody responses and protective immunity.

## References

[1] McCullers JA, Tuomanen EI (2001) Molecular pathogenesis of pneumococcal pneumonia. Front Biosci 6: D877–89. 11502489 10.2741/mccullers

[2] Briles DE, Paton JC, Hollingshead S (2009) Pneumococcal common proteins and other vaccine strategies. In: Levine MM, Kaper JB, Rappuoli R, Liu M, Good MF, New Generation Vaccines. New York, USA: Marcel Dekker. pp. 908–933.

[3] Brueggemann AB, Pai R, Crook DW, Beall B (2007) Vaccine escape recombinants emerge after pneumococcal vaccination in the United States. PLoS Pathog 3: e168. 18020702 10.1371/journal.ppat.0030168 PMC2077903

[4] Hicks LA, Harrison LH, Flannery B, Hadler JL, Schaffner W, et al (2007) Incidence of pneumococcal disease due to non-pneumococcal conjugate vaccine (PCV7) serotypes in the United States during the era of widespread PCV7 vaccination, 1998–2004. J Infect Dis 196: 1346–1354. 5. Hava DL, Camilli A (2002) Large-scale identification of serotype 4 *Streptococcus pneumoniae* virulence factors. Mol Microbiol 45: 1389–1406.

[5] Orihuela CJ, Radin JN, Sublett JE, Gao G, Kaushal D, et al (2004) Microarray analysis of pneumococcal gene expression during invasive disease. Infect Immun 72: 5582–5596. 15385455 10.1128/IAI.72.10.5582-5596.2004 PMC517545

[6] Giefing C, Meinke AL, Hanner M, Henics T, Bui MD, et al (2008) Discovery of a novel class of highly conserved vaccine antigens using genomic scale antigenic fingerprinting of pneumococcus with human antibodies. J Exp Med 205: 117–131. 18166586 10.1084/jem.20071168 PMC2234372

[7] Ogunniyi AD, Mahdi LK, Trappetti C, Verhoeven N, Mermans D, et al (2012) Identification of genes that contribute to pathogenesis of invasive pneumococcal disease by in vivo transcriptomic analysis. Infect Immun 80: 3268–3278. 22778095 10.1128/IAI.00295-12 PMC3418729

[8] Ogunniyi AD, Grabowicz M, Briles DE, Cook J, Paton JC (2007) Development of a vaccine against invasive pneumococcal disease based on combinations of virulence proteins of *Streptococcus pneumoniae* . Infect Immun 75: 350–357. 17088353 10.1128/IAI.01103-06 PMC1828427

[9] Plumptre CD, Ogunniyi AD, Paton JC (2012) Polyhistidine triad proteins of pathogenic streptococci. Trends Microbiol 20: 485–493. 22819099 10.1016/j.tim.2012.06.004

[10] Adamou JE, Heinrichs JH, Erwin AL, Walsh W, Gayle T, et al (2001) Identification and characterization of a novel family of pneumococcal proteins that are protective against sepsis. Infect Immun 69: 949–958. 11159990 10.1128/IAI.69.2.949-958.2001 PMC97974

[11] Rioux S, Neyt C, Di Paolo E, Turpin L, Charland N, et al (2010) Transcriptional regulation, occurrence and putative role of the Pht Family of *Streptococcus pneumoniae* . Microbiology 157: 336–348. 20966093 10.1099/mic.0.042184-0

[12] Zhang Y, Masi AW, Barniak V, Mountzouros K, Hostetter MK, et al (2001) Recombinant PhpA protein, a unique histidine motif-containing protein from *Streptococcus pneumoniae*, protects mice against intranasal pneumococcal challenge. Infect Immun 69: 3827–3836. 11349048 10.1128/IAI.69.6.3827-3836.2001 PMC98401

[13] Hamel J, Charland N, Pineau I, Ouellet C, Rioux S, et al (2004) Prevention of pneumococcal disease in mice immunized with conserved surface-accessible proteins. Infect Immun 72: 2659–2670. 15102774 10.1128/IAI.72.5.2659-2670.2004 PMC387903

[14] Beghetto E, Gargano N, Ricci S, Garufi G, Peppoloni S, et al (2006) Discovery of novel *Streptococcus pneumoniae* antigens by screening a whole-genome lambda-display library. FEMS Microbiol Lett 262: 14–21. 16907734 10.1111/j.1574-6968.2006.00360.x

[15] Godfroid F, Hermand P, Verlant V, Denoël P, Poolman JT (2011) Preclinical evaluation of the Pht proteins as potential cross-protective pneumococcal vaccine antigens. Infect Immun 79: 238–245. 20956575 10.1128/IAI.00378-10 PMC3019885

[16] Denoël P, Philipp MT, Doyle L, Martin D, Carletti G, et al (2011) A protein-based pneumococcal vaccine protects rhesus macaques from pneumonia after experimental infection with *Streptococcus pneumoniae* . Vaccine 29: 5495–5501. 21624422 10.1016/j.vaccine.2011.05.051 PMC5061031

[17] Bologa M, Kamtchoua T, Hopfer R, Sheng X, Hicks B, et al (2012) Safety and immunogenicity of pneumococcal protein vaccine candidates: monovalent choline-binding protein A (PcpA) vaccine and bivalent PcpA-pneumococcal histidine triad protein D vaccine. Vaccine 30: 7461–7468. 23123106 10.1016/j.vaccine.2012.10.076

[18] Seiberling M, Bologa M, Brookes R, Ochs M, Go K, et al (2012) Safety and immunogenicity of a pneumococcal histidine triad protein D vaccine candidate in adults. Vaccine 30: 7455–7460. 23131206 10.1016/j.vaccine.2012.10.080

[19] Loisel E, Chimalapati S, Bougault C, Imberty A, Gallet B, et al (2011) Biochemical characterization of the histidine triad protein PhtD as a cell surface zinc-binding protein of pneumococcus. Biochemistry 50: 3551–3558. 21425866 10.1021/bi200012f

[20] Plumptre CD, Ogunniyi AD, Paton JC (2013) Surface association of Pht proteins of *Streptococcus pneumoniae*. Infect Immun. In press. 10.1128/IAI.00562-13 PMC3811752 23876799

[21] Cognet I, De Coignac AB, Magistrelli G, Jeannin P, Aubry JP, et al (2003) Expression of recombinant proteins in a lipid A mutant of Escherichia coli BL21 with a strongly reduced capacity to induce dendritic cell activation and maturation. J Immunol Methods 272: 199–210. 12505724 10.1016/s0022-1759(02)00506-9

[22] LeMessurier KS, Ogunniyi AD, Paton JC (2006) Differential expression of key pneumococcal virulence genes in vivo. Microbiology 152: 305–311. 16436418 10.1099/mic.0.28438-0

[23] Lupas A, Van Dyke M, Stock J (1991) Predicting coiled coils from protein sequences. Science 252: 1162–1164. 2031185 10.1126/science.252.5009.1162

[24] Compton LA, Johnson WC (1986) Analysis of protein circular dichroism spectra for secondary structure using a simple matrix multiplication. Anal Biochem 155: 155–167. 3717552 10.1016/0003-2697(86)90241-1

[25] Whitmore L, Wallace BA (2004) DICHROWEB, an online server for protein secondary structure analyses from circular dichroism spectroscopic data. Nucleic Acids Res 32: W668–73. 15215473 10.1093/nar/gkh371 PMC441509

[26] Whitmore L, Wallace BA (2008) Protein secondary structure analyses from circular dichroism spectroscopy: methods and reference databases. Biopolymers 89: 392–400. 17896349 10.1002/bip.20853

[27] McDaniel LS, Ralph BA, McDaniel DO, Briles DE (1994) Localization of protection-eliciting epitopes on PspA of *Streptococcus pneumoniae* between amino acid residues 192 and 260. Microb Pathog 17: 323–337. 7723659 10.1006/mpat.1994.1078

[28] Briles DE, Hollingshead SK (2006) Surface proteins of *Streptococcus pneumoniae*: their roles in virulence and potential as vaccines. Program and abstracts of the 2006 Euroconference on Infections and Lung Diseases. Paris, France.

[29] Malley R, Anderson PW (2012) Serotype-independent pneumococcal experimental vaccines that induce cellular as well as humoral immunity. Proc Natl Acad Sci USA 109: 3623–3627. 22308483 10.1073/pnas.1121383109 PMC3309758

[30] Ogunniyi AD, Folland RL, Briles DE, Hollingshead SK, Paton JC (2000) Immunization of mice with combinations of pneumococcal virulence proteins elicits enhanced protection against challenge with *Streptococcus pneumoniae* . Infect Immun 68: 3028–3033. 10769009 10.1128/iai.68.5.3028-3033.2000 PMC97524

[31] Kadioglu A, Andrew PW (2005) Susceptibility and resistance to pneumococcal disease in mice. Brief Funct Genomic Proteomic 4: 241–247. 16420749 10.1093/bfgp/4.3.241

[32] Khan MN, Pichichero ME (2013) CD4 T cell memory and antibody responses directed against pneumococcal histidine triad proteins PhtD and PhtE following nasopharyngeal colonization and immunization and their role in protection against pneumococcal colonization in mice. Infect Immun. In press. 10.1128/IAI.00313-13 PMC3811773 23897609

[33] Paton JC, Lock RA, Lee CJ, Li JP, Berry AM, et al (1991) Purification and immunogenicity of genetically obtained pneumolysin toxoids and their conjugation to *Streptococcus pneumoniae* type 19F polysaccharide. Infect Immun 59: 2297–2304. 2050399 10.1128/iai.59.7.2297-2304.1991 PMC258010

